# Elucidating the role of keratin 75 in enamel using Krt75^tm1Der^ knock-in mouse model

**DOI:** 10.3389/fphys.2022.1102553

**Published:** 2022-12-23

**Authors:** Rutuja Deshmukh, Brent Vasquez, Lasya Bhogadi, Claire M. Gabe, Lyudmila Lukashova, Kostas Verdelis, Maria I. Morasso, Elia Beniash

**Affiliations:** ^1^ Center for Craniofacial Regeneration, Pittsburgh, PA, United States; ^2^ Department of Oral and Craniofacial Sciences, University of Pittsburgh School of Dental Medicine (UPSDM), Pittsburgh, PA, United States; ^3^ Department of Endodontics, University of Pittsburgh School of Dental Medicine (UPSDM), Pittsburgh, PA, United States; ^4^ Laboratory of Skin Biology, National Institute of Arthritis and Musculoskeletal and Skin Diseases, Bethesda, MD, United States

**Keywords:** keratin, ameloblast, enamel matrix, dental caries, biomineralization

## Abstract

Keratin 75 (K75) was recently discovered in ameloblasts and enamel organic matrix. Carriers of A161T substitution in K75 present with the skin condition *Pseudofollicullitis barbae*. This mutation is also associated with high prevalence of caries and compromised structural and mechanical properties of enamel. Krt75^tm1Der^ knock-in mouse (KI) with deletion of Asn159, located two amino acids away from KRT75^A161T^, can be a potential model for studying the role of K75 in enamel and the causes of the higher caries susceptibility associated with KRT75^A161T^ mutation. To test the hypotheses that KI enamel is more susceptible to a simulated acid attack (SAA), and has altered structural and mechanical properties, we conducted *in vitro* SAA experiments, microCT, and microhardness analyses on 1st molars of one-month-old WT and KI mice. KI and WT hemimandibles were subjected to SAA and contralateral hemimandibles were used as controls. Changes in enamel porosity were assessed by immersion of the hemimandibles in rhodamine, followed by fluorescent microscopy analysis. Fluorescence intensity of KI enamel after SSA was significantly higher than in WT, indicating that KI enamel is more susceptible to acid attack. MicroCT analysis of 1st molars revealed that while enamel volumes were not significantly different, enamel mineral density was significantly lower in KI, suggesting a potential defect of enamel maturation. Microhardness tests revealed that in KI enamel is softer than in WT, and potentially less resilient to damages. These results suggest that the KI enamel can be used as a model to study the role of K75 in enamel.

## Introduction

Dental enamel, the hardest and most highly mineralized tissue, comprises the outer layer of tooth crowns. Enamel is the only mineralized tissue of epithelial origin and the only acellular mineralized tissue which is incapable of remodeling or repair. Despite of this limitation it can withstand millions of cycles of mastication over the lifetime without catastrophic damage due to its unique combination of high hardness and fracture toughness. These mechanical properties of enamel are determined by its unique hierarchical structure and composition (Cuy et al., 2002; Chai et al., 2009; He and Swain, 2009; Yilmaz et al., 2015; Beniash et al., 2019; DeRocher et al., 2020; Wilmers and Bargmann, 2020). The major component of enamel is mineral, carbonated hydroxyapatite, comprising ∼95% of this tissue by weight. Extracellular organic matrix (ECM) of the mature enamel accounts for roughly 1% by weight with the rest being water. Despite the fact that in mature enamel ECM is a minor component, it plays an important toughening role (Baldassarri et al., 2008; Yahyazadehfar and Arola, 2015). The basic building block of enamel is the enamel rod—a bundle of high aspect ratio crystallites. Individual enamel rods run from the dentin-enamel junction (DEJ) to the enamel surface. The tracks of enamel rods are form a complex decussating pattern (Wilmers and Bargmann, 2020). The rods are embedded in an interrod matrix consisting of crystals oriented at an angle to the rod crystals (Beniash et al., 2019).

Enamel mineral is susceptible to dissolution under acidic conditions. Bacteria in enamel plaque, the biofilm on the enamel surface, produce lactic acid, which dissolves enamel mineral, leading to formation of caries lesions (Featherstone, 2000). These lesions, if left untreated, can lead to weakening of the tooth, inflammation of the pulp tissues and tooth loss. Caries is the most prevalent chronic infectious disease of the oral cavity affecting 60%–90% of children and the vast majority of adults (Petersen et al., 2005) and it is well established that genetic factors affect susceptibility to caries (Shuler, 2001; Shaffer et al., 2011; Vieira et al., 2014). At the same time, due to the multifactorial etiology, the relationships between individual polymorphisms and caries susceptibility are often weak, although a number of genes related to odontogenesis and antibacterial defense show significant associations (Vieira et al., 2014).

Although mature enamel is the most highly mineralized tissue it starts as a self-assembled protein gel (Margolis et al., 2006) in which the nascent mineral crystalline ribbons are suspended. The secretion of enamel is carried out by ameloblasts, highly specialized secretory cells of the epithelial origin (Lacruz et al., 2017). Secretory ameloblasts form a highly specialized secretory apparatus—Tomes’ process, responsible for secretion of one rod and a portion of the interrod matrix. During the secretory stage ameloblasts move away from the DEJ depositing enamel rods. The secreted enamel matrix consists of three major enamel matrix proteins (EMPs)–amelogenin, comprising ∼90% of the ECM, ameloblastin and enamelin (Margolis et al., 2006). EMPs undergo a series of proteolytic cleavages, which are essential for the proper enamel formation (Simmer and Hu, 2002). During the maturation phase, the mineral crystals grow in thickness and EMPs are degraded and removed (Pham et al., 2017). At the end of the maturation only a small organic fraction containing small peptides and insoluble proteinaceous matrix remains. This small fraction of heavily crosslinked proteins in mature enamel exists in a form of enamel tufts and rod sheaths (Amizuka et al., 1992). The composition of this insoluble proteinaceous matrix remains poorly understood (Duverger et al., 2016). It was established that a small fraction of enamel matrix deposited during secretory stage of amelogenesis remains, while the majority of transient proteins, collectively called amelogenins at this time, is degraded (Robinson et al., 1971; Robinson et al., 1975, 1977). It has been proposed that this insoluble fraction contains keratins (Robinson et al., 1989) and is heavily cross-linked *via* isopeptide bonds (Robinson and Hudson, 2011). However, until recently the evidence of keratins in enamel was sparse and inconclusive.

Recently a number of epithelial keratins were identified in the enamel organ using RNA-Seq (Duverger et al., 2014). One of these keratins–keratin 75 (K75, formerly K6hf) has a highly specific expression pattern. It is expressed in the inner sheath of the hair follicle, nail bed and lingual papillae (Winter et al., 1998; Wang et al., 2003). More recently, KRT75 was identified in ameloblasts and enamel ECM by immunochemistry, western blot and mass spectrometry (Duverger et al., 2014; Chiba et al., 2019; Yang et al., 2019). A single amino acid substitution KRT75^A161T^, caused by A to G missense mutation in KRT75 gene is associated with *Pseudofolliculitis barbae* (PB) colloquially known as barber rash (Duverger et al., 2014). This gene mutation leads to Ala161 to Thr substitution at the beginning of the highly conserved A1 α-helical region of the protein. Interestingly, the same polymorphism is also associated with the higher prevalence of caries and altered structural and mechanical properties of dental enamel. Importantly, western blot analysis of forming porcine enamel revealed that, unlike other EMPs, K75 is not degraded during the maturation stage. Several other mutations in conserved regions epithelial keratins expressed in ameloblasts have also been found to be associated with higher caries susceptibility and enamel anomalies (Duverger et al., 2018; Duverger et al., 2019; Raza et al., 2022).

There are currently no animal models of PB, however Krt75^tm1Der^ knock-in (KI) mouse with deletion of Asn159, a highly conserved amino acid in the α-helix initiation motif is available (Chen et al., 2008). Asn159 is only 2 amino acids away from Ala161, which when substituted by Thr is associated with higher caries susceptibility (Duverger et al., 2014). We hypothesize that the conserved region around Ala161 is critical for K75 function and that mice with Asn159 deletion will have altered enamel properties and higher caries susceptibility. To test this hypothesis, we conducted a comparative study of molar enamel in WT and Krt75^tm1Der^ mice using Microcomputed Tomography (μCT), Light Microscopy, simulated acid attack, and microhardness (MH) analysis.

## Materials and methods

### Animal model

Krt75^tm1Der^ KI mouse on the C57BL/6 background with deletion of 159Asn, two amino acids away from A161T substitution site were used for this study. The mice are housed at the University of Pittsburgh DLAR facility and all the studies were approved by the University of Pittsburgh IACUC. Mice were euthanized using carbon dioxide and secondary euthanasia was performed. Prior to analysis, hemimandibles from 1-month old WT and KI mice were dissected and stored immediately in 70% ethanol.

### μCT analysis

For μCT analysis right hemi-mandibles of 1-month-old mice of WT and homozygous Krt75^tm1Der^ genotype were used. Four animals per group were used in the study. The mandibles were dissected, cleaned from soft tissues and kept in 70% ethanol. Microcomputed tomography (µCT) analysis was performed on Scanco µCT 50 (Scanco Medical, Brüttisellen, Switzerland) system. The hemimandibles were scanned at 6 µm voxel size, 55 KVp, 0.36 rotation step (180°angular range) and a 1500 m exposure per view. The scans were performed in 70% EtOH. The Scanco µCT software (HP, DEC windows Motif 1.6) was used for 3D reconstruction and viewing of the images. Quantitative analysis was performed on an operator-defined region of interest around the crowns of 1st molars of right hemimandibles. Volumes within the region of interest were segmented using a global threshold of 0.84 g HA/cc and total enamel volume and mineral density of enamel of the first molars were calculated.

### Microhardness tests

Microhardness tests were performed on left hemimandibles from the animals used for μCT analysis. The hemimandibles were air dried and mounted in Epoxy resin (EMS, Hatfield, PA). The samples were ground using Minimet 1000 semi-automatic grinder-polisher (Buehler, Lake Bliff, IL) on the mesio-distal plane until the enamel from the first molars was exposed using 400 grit paper. The samples then were polished using 600 and 800 grit papers, followed by final polishing with 12, 6, 1, and 0.5 μm MetaDi diamond suspensions (Buehler, Lake Bluff, Il). The micro indentation test was then performed using IdentaMet 1105 microhardness tester (Buehler, Lake Bluff, Il) equipped with a CCD camera, connected to a computer equipped with OmniMet software (Buehler, Lake Bluff, Il). The tests were conducted using a Vickers hardness diamond tip with a load of 25 gf and dwell time of 5 s. Six measurements in the outer and 6 measurements in the inner enamel were taken from the mesial enamel of the first molar of each specimen and the averages of those 6 measurements were used the Vickers hardness numbers (HV) for the outer and inner enamel.

### Simulated acid attack experiments

Intact right hemimandibles from 4 1-month-old mice of each genotype were used in the simulated acid attack experiments. The experiments were designed based on the published procedure (Vieira et al., 2015). Each mandible was mounted in a Petri dish using double sided tape and were submerged in 40 ml of the demineralization solution containing 1.3 mmol/L Ca, 0.78 mmol/L P, 0.05 mol/L acetate buffer, and 0.03 ugF/ml, pH 5.0 to induce artificial caries (Vieira et al., 2015). The teeth were exposed to the demineralizing solution for 10 h, rinsed in distilled deionized water (DDW) for 5 min followed by incubation in 2 changes of Ca^2+^-free PBS for 5 min each. Left non-treated hemimandibles from the same animals were used as controls.

### Rhodamine staining

Hemimandibles exposed to demineralizing solutions and the contralateral controls were incubated in 0.02% Rhodamine-6G in PBS for 30 min. The mandibles were rinsed in PBS on a rocking table for 5 min. The mandibles then were microphotographed using a fluorescent dissecting microscope Leica DFC 450 under the same light conditions and exposure. Micrographs of buccal, lingual and occlusal surfaces of molars were taken per each specimen. The micrographs were analyzed using Fiji (ImageJ) image processing software package. The areas of 1st molars were selected from the buccal views of all specimens and the average fluorescence intensities were calculated. The statistical analysis of the data was performed using OriginPro 2017 graphing and data analysis software package.

### Statistical analysis

Statistical analyses of the data were performed using OriginPro 2017 graphing and data analysis software package. The data was analyzed using one-way ANOVA and t-tests in which equal variance was not assumed (Welch correction). Statistically significant differences were determined at *p*-value ≤ 0.05.

## Results

### μCT analysis of molar enamel

**FIGURE 1:**
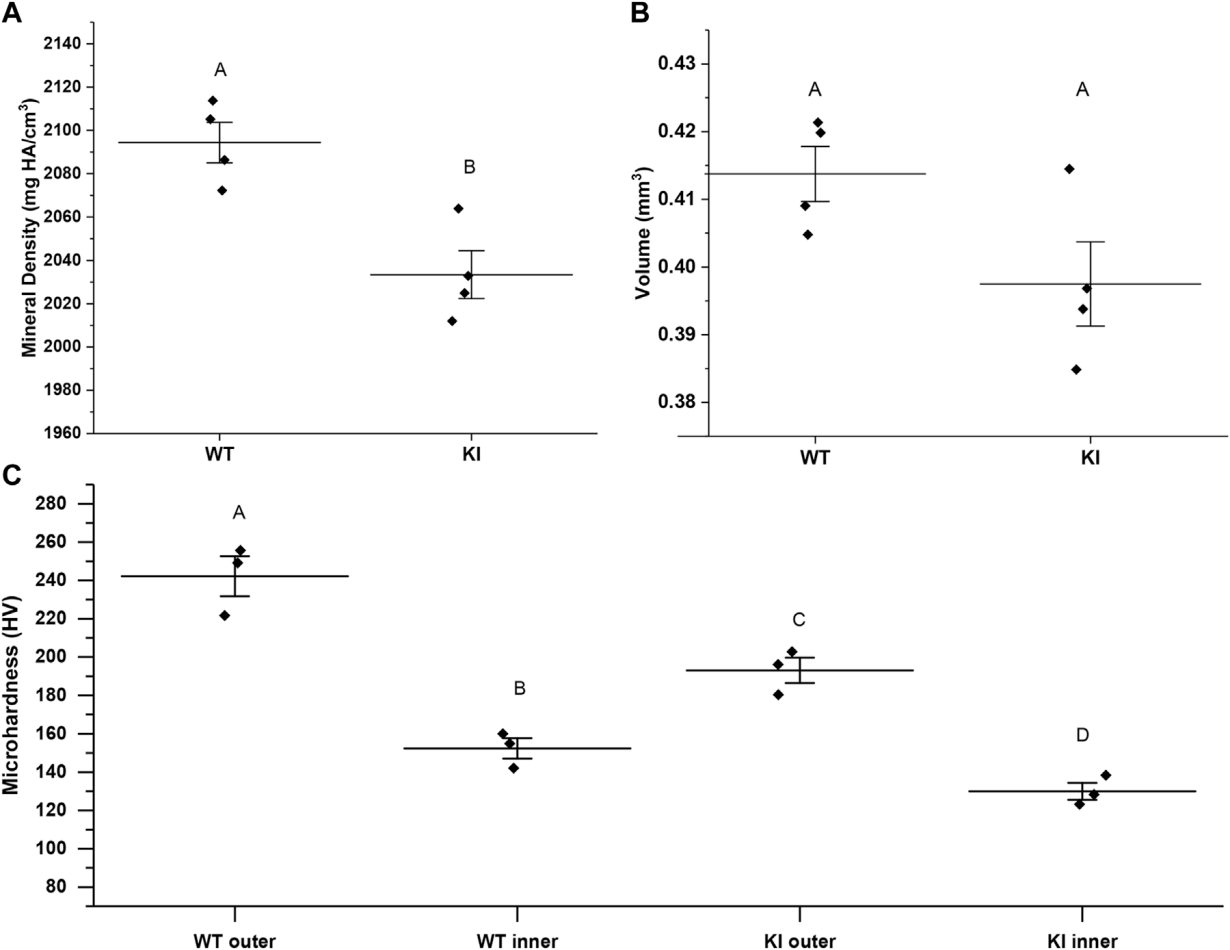
MicroCT analysis of mineral density **(A)** and volume **(B)** of enamel from 1 month-old WI and KI mice. **(C)** Microhardness values for 1-month-old WT and KI 1st molar enamel. Diamonds represent values of individual specimens. Horizontal lines indicate means and whiskers show standard errors. Similar letters indicate populations which are not significantly different from each other.

Mineral density (MD) and enamel volume (EV) data from the 1st molar enamel from 1-month-old WT (*n* = 4) and KI mice (*n* = 4) were collected and analyzed. MD of enamel from KI mice was significantly lower than that of WT (2033.42, SD = 22.07 vs. 2094.37 ± 18.69; *p* = 0.006) (Figure 1A). There were no significant differences in EV between two genotypes (Figure 1B).

### Microhardness studies

Two-way ANOVA revealed that the population means were significantly different between genotypes and between outer and inner enamel (*p* < 0.01 for both). Microhardness of inner enamel was significantly lower than that of outer enamel in both genotypes (*p* = 0.001 for WT and *p* = 0.02 for KI) (Figure 1C; Table 1). Microhardness of outer enamel was significantly lower in KI than in WT (*p* = 0.02). Similarly, microhardness of inner enamel was significantly lower in KI (*p* = 0.03) (Figure 1C; Table 1).

**TABLE 1:** Microhardness values (HV) of 1-month-old 1st molars.

	N	Mean	St. Dev	Min	Median	Max
WT outer	3	246.5	27.15	215.4	259.0	265.2
WT inner	3	153.5	19.14	137.7	148.1	174.8
KI outer	3	185.9	13.20	170.7	193.2	193.9
KI inner	3	127.6	4.526	122.5	128.8	131.3

### Simulated acid attack experiments

**FIGURE 2:**
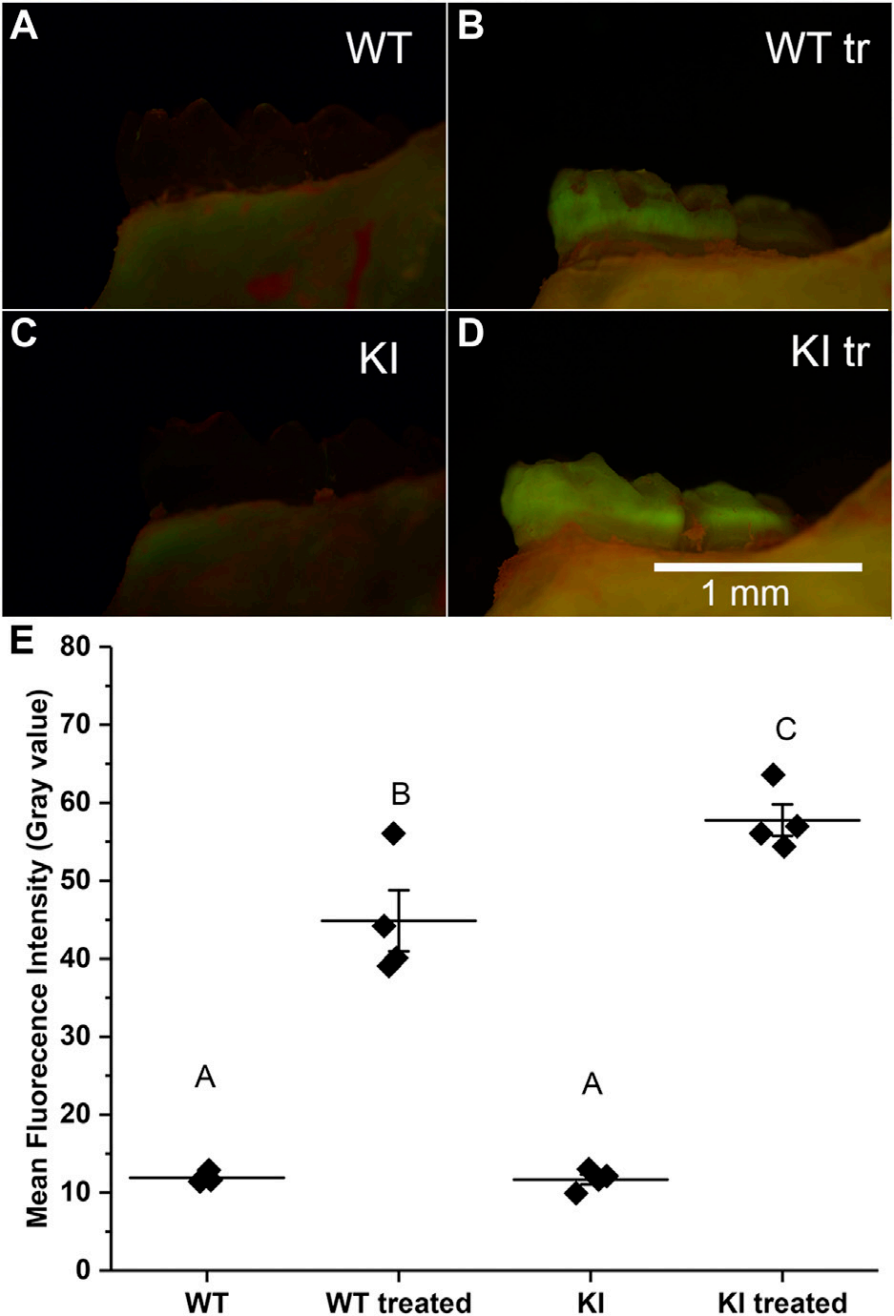
Micrographs of hemimandibles in buccal view of **(A)** untreated WT, **(B)** treated WT, **(C)** untreated KI, **(D)** treated KI. **(E)** A plot of mean fluorescence intensity (grey values) obtained for buccal surfaces of 1st molars. Diamonds represent mean fluorescence intensity for each specimen. Horizontal lines represent mean values for each group and whiskers indicate standard errors. Similar letters indicate populations which are not significantly different from each other.

The acid attack experiments were conducted as described in Materials and methods. The extent of demineralization was determined by changes in the fluorescence intensity of the samples after exposure to Rhodamine-6G. We hypothesized that enamel of teeth with more extensive demineralization will retain more rhodamine-6G due to the higher porosity and will therefore have higher fluorescence intensity, as has been shown previously in other systems (Lee et al., 2003; Ebacher and Wang, 2009). Our observations revealed that untreated samples had much lower fluorescence intensity than the treated samples in both groups (Figures 2A–D). Furthermore, our visual examination has strongly suggested that KI treated molars had higher levels of fluorescence than WT treated molars (Figures 2B, D).

To assess quantitative differences in average intensities (grey level) among four groups we conducted a two-way ANOVA test which showed that population means of treatment and genotype are significantly different (*p* < 0.01). The *post hoc* Bonferroni test revealed that the intensity in control specimens was substantially and significantly lower than in the treatment groups (*p* < 0.0001 in both cases) and the mean intensity values of control specimens were not significantly different (Figure 2E; Table 2). The mean intensity of KI 1st molars exposed to the simulated acid attack was significantly higher than of WT 1st molars (*p* < 0.04) suggesting that the Krt75^tm1Der^ KI mice are significantly more susceptible to acid attack.

**TABLE 2:** Rhodamine Fluorescence Intensity (gray values) of 1-month-old 1st molars.

	N	Mean	St. Dev	Min	Max
WT	4	11.9	0.66	11.4	12.9
WT treated	4	44.9	7.79	39.1	56.1
KI	4	11.7	1.30	9.9	13.0
KI treated	4	57.8	4.03	54.4	63.6

## Discussion

Our studies revealed significant differences in several physical and chemical characteristics of enamel of 1st molars from 1-month old WT and Krt75^tm1Der^ KI mice. Our µCT data showed a small but highly significant decrease in enamel density. The fact that enamel in 1-month-old KI animals is less dense than that in WT of the same age potentially points out to enamel maturation problems in KI, since at this age the crowns are exposed to the environment for less than 2 weeks and it is unlikely that the lower density is due to leaching of mineral ions. At the same time, there were no differences in the volume of enamel in both genotypes and in its overall appearance. This parallels the situation in human carriers of KRT75^A161T^ polymorphism, in which the clinical appearance of the teeth is normal, however the quality of enamel is altered (Duverger et al., 2014).

Our microhardness studies of 1st molars in 1-month-old WT and KI mice demonstrate the reduced hardness of enamel in KI. These results also parallel the results of the microhardness studies of enamel in humans, which show a reduced enamel hardness in carriers of A161T substitution in K75. However in humans the reduction in hardness was only observed in the inner enamel (Duverger et al., 2014). These differences can be attributed to the differences in decussating pattern between human and rodent molar enamel. The reduction in enamel hardness can potentially lead to higher enamel wear, and increased susceptibility to accumulating microdamages.

The results of the simulated acid attack revealed a greater increase in porosity of KI enamel after the treatment, which indicates KI enamel is more soluble. Again, these results parallel the observations that KRT75^A161T^ polymorphism in humans which is associated to higher susceptibility to acid attack (Duverger et al., 2014).

Overall, these results indicate that enamel properties on Krt75^tm1Der^ KI to a large degree recapitulate the altered properties of enamel in human carriers of KRT75^A161T^ polymorphism, and that this KI can be used as a model to study the effects of point mutations in conserved regions of epithelial keratins expressed by ameloblasts on enamel properties and the susceptibility to caries. Such future studies can provide insights into roles of keratins in enamel formation and its function and will lead to a better understanding of genetic basis of caries susceptibility.

## Data Availability

The original contributions presented in the study are included in the article/Supplementary Materials, further inquiries can be directed to the corresponding author.
